# Multiple Mechanistically Distinct Timescales of Neocortical Plasticity Occur During Habituation

**DOI:** 10.3389/fncel.2022.840057

**Published:** 2022-04-08

**Authors:** Francesca A. Chaloner, Sam F. Cooke

**Affiliations:** ^1^MRC Centre for Neurodevelopmental Disorders (CNDD), King’s College London, London, United Kingdom; ^2^Department of Basic and Clinical Neuroscience, King’s College London, London, United Kingdom

**Keywords:** primary visual cortex, learning, adaptation, habituation, inhibition, novelty, stimulus-selective response potentiation, NMDA receptors

## Abstract

Recognizing familiar but innocuous stimuli and suppressing behavioral response to those stimuli are critical steps in dedicating cognitive resources to significant elements of the environment. Recent work in the visual system has uncovered key neocortical mechanisms of this familiarity that emerges over days. Specifically, exposure to phase-reversing gratings of a specific orientation causes long-lasting stimulus-selective response potentiation (SRP) in layer 4 of mouse primary visual cortex (V1) as the animal’s behavioral responses are reduced through habituation. This plasticity and concomitant learning require the NMDA receptor and the activity of parvalbumin-expressing (PV+) inhibitory neurons. Changes over the course of seconds and minutes have been less well studied in this paradigm, so we have here characterized cortical plasticity occurring over seconds and minutes, as well as days, to identify separable forms of plasticity accompanying familiarity. In addition, we show evidence of interactions between plasticity over these different timescales and reveal key mechanistic differences. Layer 4 visual-evoked potentials (VEPs) are potentiated over days, and they are depressed over minutes, even though both forms of plasticity coincide with significant reductions in behavioral response. Adaptation, classically described as a progressive reduction in synaptic or neural activity, also occurs over the course of seconds, but appears mechanistically separable over a second as compared to tens of seconds. Interestingly, these short-term forms of adaptation are modulated by long-term familiarity, such that they occur for novel but not highly familiar stimuli. Genetic knock-down of NMDA receptors within V1 prevents all forms of plasticity while, importantly, the modulation of short-term adaptation by long-term familiarity is gated by PV+ interneurons. Our findings demonstrate that different timescales of adaptation/habituation have divergent but overlapping mechanisms, providing new insight into how the brain is modified by experience to encode familiarity.

## Significance Statement

Habituation is a foundational cognitive process that reduces the requirement for neural resources to be allocated to innocuous stimuli, thereby freeing up attention and energy to detect and explore salience. Memories of innocuous familiar stimuli must be formed so that they can be selectively ignored while novel stimuli, which have the potential for significance, are detected. Within the visual system, we have previously shown that increases in neural activity in cerebral cortex occur during habituation that emerges over days, but many forms of habituation must occur over shorter timescales to allow allocation of resources to appropriate stimuli within a single session. Here we characterize cortical plasticity and habituation over seconds, minutes, and days within the same subjects, revealing short-term plasticity that diminishes neural activity, an opposing effect to the better characterized long-term plasticity. In addition, we have revealed overlapping but distinct molecular and cellular mechanisms mediating these different timescales of plasticity. Elucidating the mechanisms that underlie habituation will inform us how the brain can learn to recognize familiar stimuli and thereby detect novelty. This work also provides unique insight into core processes of learning that are affected in the disordered brain, where habituation and novelty detection are commonly dysfunctional.

## Introduction

Learning and memory enable organisms to adapt to altered pressures in the environment to produce appropriate responses to stimulus and context over a variety of timescales (McGaugh, 2000). Substantial gaps remain in our understanding of the neural underpinnings of these processes, in part due to difficulties in observing and intervening in underlying plasticity as learning and memory occur (Neves et al., 2008). Habituation is one relatively robust, easy to observe and apparently simple form of learning, in which organisms acquire familiarity with innocuous stimuli and selectively reduce behavioral responses to those stimuli over seconds, minutes, and days (Cooke and Ramaswami, 2020). Habituation forms a foundation for further learning by enabling energy and attention to be devoted to stimuli of already established salience, or novel stimuli that may have future significance (Rankin et al., 2009; Schmid et al., 2014) and disruptions in this process likely contribute to a range of psychiatric and neurological disorders (Ramaswami, 2014; McDiarmid et al., 2017). This form of learning has commonly been ascribed to a neural process known as adaptation, which reduces feedforward synaptic activity in response to repeated non-associative stimulation (Groves and Thompson, 1970), especially over shorter timescales (Chung et al., 2002). However, a competing theory, known as the comparator model (Sokolov, 1963), suggests the formation of long-lasting memory of familiar stimuli through Hebbian synaptic potentiation, which in turn suppresses behavioral output by recruiting inhibitory systems. It remains possible that both models apply but over different timescales (Cooke and Ramaswami, 2020). In this study, we have assessed plasticity in primary visual cortex (V1) of mice in response to repeated presentations of oriented, phase reversing visual stimuli to assess whether different directions of plasticity can be observed across different timescales.

It is now well established that the magnitude of visual-evoked potentials (VEPs) recorded in layer 4 of mouse binocular V1 increases dramatically over days of repeated stimulation through an orientation-specific form of plasticity known as stimulus-selective response potentiation (SRP) (Frenkel et al., 2006; Cooke and Bear, 2010). This form of plasticity is also manifest as an increase in the peak firing rate of V1 neurons (Aton et al., 2014; Cooke et al., 2015) and many of the known molecular mechanisms are consistent with the involvement of Hebbian synaptic potentiation, notably including a requirement for the NMDA receptor during induction and AMPA receptor insertion during expression (Frenkel et al., 2006; Cooke and Bear, 2010). Importantly, mice produce behavioral responses to the onset of these visual stimuli that exhibit significant orientation-selective habituation over days (Cooke et al., 2015; Kaplan et al., 2016; Fong et al., 2020; Finnie et al., 2021), and this process also requires the presence of NMDA receptors in V1. In addition, a cortical cell-type that exerts exquisite inhibitory control over excitatory cell activity, the parvalbumin-expressing (PV+) inhibitory interneurons (Atallah et al., 2012), are critical for differential cortical and behavioral responses to familiar and novel stimuli after SRP and accompanying habituation (Kaplan et al., 2016). Thus, SRP comprises a robust and relatively well understood form of plasticity that occurs concomitantly with and shares mechanism with long-term memory.

One fascinating feature of SRP is that it does not manifest within a ∼30-min recording session but starts to emerge the following day (Frenkel et al., 2006) and recent work has demonstrated that SRP is dependent on consolidation processes that occur during sleep (Aton et al., 2014; Durkin et al., 2017). Activity in the primary visual relay nucleus of the thalamus, the dorsal lateral geniculate nucleus (dLGN), does increase over the course of 30 min prior to the emergence of SRP in the cortex (Durkin et al., 2017), but there has so far been no description of what happens over this time-course in V1. Although we have previously described evidence for a faster adaptation that is apparent when comparing the beginning of a 200-phase reversal block with the end (Kim et al., 2020), we have not described the time-course of this adaptation during this 100-s block. In neither case is there any understanding of the underlying mechanism. In the current study, we show that cortical plasticity accompanying behavioral habituation occurs across seconds, minutes, and days of repeated stimulus experience. Notably, these forms of plasticity diverge in direction and mechanism, and there is evidence of an interaction in which long-term familiarity suppresses adaptation. In striking opposition to our observations of SRP during long-term habituation (Cooke et al., 2015), layer 4 response magnitude decreases over seconds and minutes in V1. Loss of expression of NMDA receptors from neurons in V1 impairs plasticity and adaptation across all timescales. However, inactivation of PV+ neurons has a more nuanced effect, revealing the existence of two separable forms of fast adaptation within a stimulus block. Moreover, we show that the interaction between long-lasting familiarity and adaptation requires the activity of PV+ neurons. Thus, a range of mechanistically separable forms of plasticity can be assayed across different timescales in the same learning mouse.

## Materials and Methods

### Animals

**FIGURE 1:**
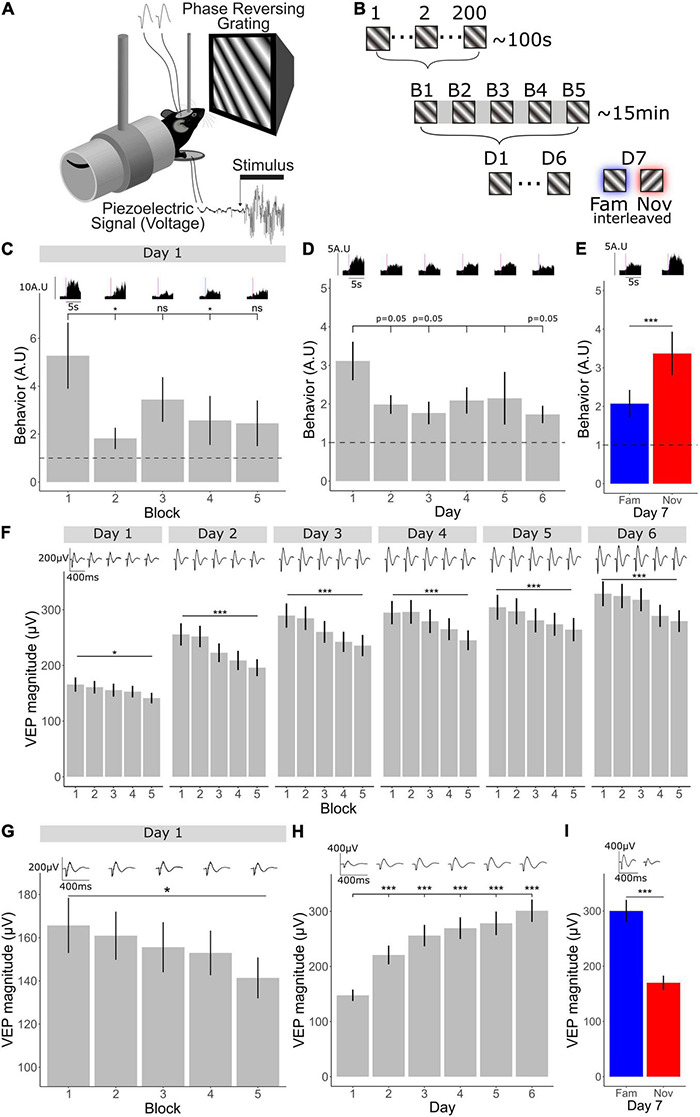
V1 plasticity accompanying long- and short-term habituation occurs in opposing directions. **(A)** Schematic of recording set-up. Mice viewed phase reversing gratings while layer 4 local-field potentials were recorded through implanted tungsten electrodes and movement was recorded through a piezo-electrical device. **(B)** 1 through 200 individual phase reversals were shown lasting approximately 100 s (1 block). Five blocks were shown lasting approximately 15 min within one session. One session of 5 blocks was shown for 6 days. On the 7th day, the familiar orientation (previously viewed) and a novel orientation were shown pseudo-randomly interleaved. **(C)** Comparison of behavior across blocks (*n* = 30). Friedman test χ^2^(4) = 13.8, *p* = 0.008. *Post-hoc* analysis of individual comparisons of blocks 1–2: *p* = 0.02, blocks 1–3: *p* = 0.7, blocks 1–4: *p* = 0.04, blocks 1–5: *p* = 0.5. FDR correction for multiple comparisons. **(D)** Behavioral change over days 1–6 (*n* = 30). Freidman test χ^2^(5) = 6.55, *p* = 0.3. *Post-hoc* analysis of individual comparisons of days 1–2: *p* = 0.05, days 1–3: *p* = 0.05, days 1–4: *p* = 0.2, days 1–5: *p* = 0.09, days 1–6: *p* = 0.05. FDR correction for multiple comparisons. **(E)** Behavioral response to familiar and novel (*n* = 30). Wilcoxon signed-rank test fam vs. nov: *p* < 0.001. **(F)** VEP magnitude from block 1 to 5 over 6 days (*n* = 33). Comparison across blocks, Friedman test, day 1: χ^2^(4) = 12.8, *p* = 0.01, day 2: χ^2^(4) = 69.8, *p* < 0.001, day 3: χ^2^(4) = 55.1, *p* < 0.001, day 4: χ^2^(4) = 43.8, *p* < 0.001, day 5: χ^2^(4) = 32.5, *p* < 0.001, day 6: χ^2^(4) = 38.6, *p* < 0.001. FDR correction for multiple comparisons. **(G)** VEP magnitude from block 1 to 5 on day 1 (n = 33). Friedman test across blocks on day 1; *p* = 0.01. **(H)** VEP magnitude potentiation over days 1–6 (*n* = 33). Freidman test χ^2^(5) = 95.9, *p* < 0.001. *Post-hoc* analysis of individual comparisons of days 1, 2, 3, 4, 5, day 6: all *p* < 0.001, FDR correction for multiple comparisons. **(I)** VEP magnitude response to familiar and novel (*n* = 33). Wilcoxon signed-rank test fam vs. nov: *p* < 0.001. Asterisks throughout denote significance (*p < 0.05, ***p < 0.001) while ns denotes non-significant. Where *p* = 0.05, this is explicitly stated.

**FIGURE 2:**
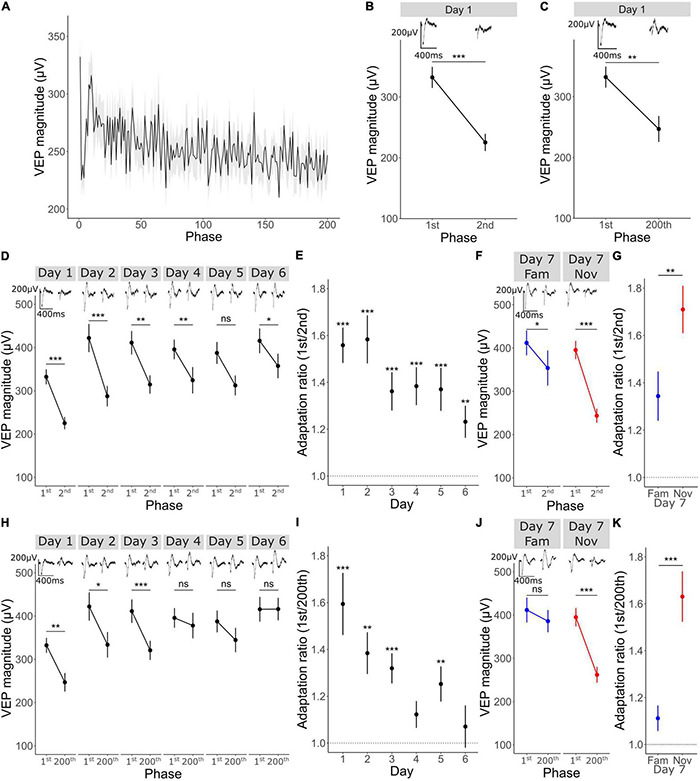
Short-term adaptation occurs within a stimulus block and is modulated by familiarity. **(A)** Mean ± SEM VEP magnitude for phase reversal 1–200 (*n* = 33). **(B)** VEP magnitude in response to the first phase reversal and the 2nd, Wilcoxon signed rank 1st vs. 2nd: *p* < 0.001 (*n* = 33). **(C)** VEP magnitude in response to the first phase reversal and the 200th, Wilcoxon signed rank 1st vs. 200th: *p* = 0.001 (*n* = 33). **(D)** VEP potential magnitude in response to the 1st vs. 2nd phase reversal over 6 days (*n* = 33). Wilcoxon signed rank 1st vs. 2nd day 1: *p* < 0.001, day 2: *p* < 0.001, day 3: *p* = 0.002, day 4: *p* = 0.008, day 5: *p* = 0.05, day 6: *p* = 0.04. FDR correction for multiple comparisons. **(E)** Adaptation ratio (1st/2nd) over 6 days. Wilcoxon signed-rank test on AR (μ = 1) day 1: *p* < 0.001, day 2: *p* < 0.001, day 3: *p* < 0.001, day 4: *p* < 0.001, day 5: *p* < 0.001, day 6: *p* = 0.002. FDR correction for multiple comparisons. **(F)** VEP potential magnitude in response to the 1st vs. 2nd phase reversal on day 7 (*n* = 33). Wilcoxon signed rank 1st vs. 2nd day 7 fam: *p* = 0.02, day 7 nov: *p* < 0.001. FDR correction for multiple comparisons. **(G)** Adaptation ratio (1st/2nd) on day 7. Wilcoxon signed-rank test fam vs. nov: *p* = 0.009. **(H)** VEP potential magnitude in response to the 1st vs. 200th phase reversal over 6 days (*n* = 33). Wilcoxon signed rank 1st vs. 200th day 1: *p* = 0.008, day 2: *p* = 0.04, day 3: *p* < 0.001, day 4: *p* = 1, day 5: *p* = 0.4, day 6: *p* = 1. **(I)** Adaptation ratio (1st/200th) over 6 days. Wilcoxon signed-rank test on AR (μ = 1) day 1: *p* < 0.001, day 2: *p* = 0.001, day 3: *p* < 0.001, day 4: *p* = 0.1, day 5: *p* = 0.006, day 6: *p* = 1. FDR correction for multiple comparisons. **(J)** VEP potential magnitude in response to the 1st vs. 2nd phase reversal on day 7 (*n* = 33). Wilcoxon signed rank 1st vs. 200th day 7 fam: *p* = 1, day 7 nov: *p* < 0.001. **(K)** Adaptation ratio (1st/200th) on day 7. Wilcoxon signed-rank test fam vs. nov: *p* < 0.001. Asterisks throughout denote significance (*p < 0.05, **p < 0.01, ***p < 0.001) while ns denotes non-significant.

**FIGURE 3:**
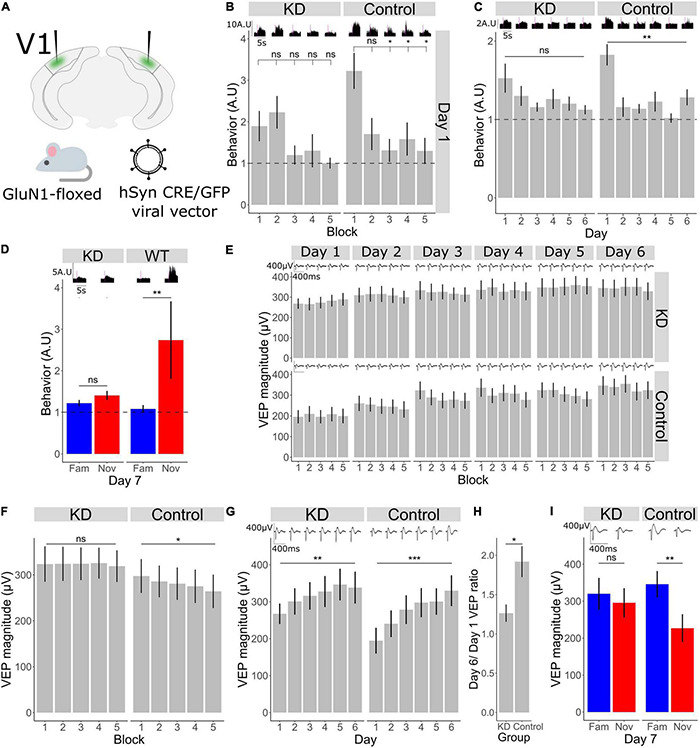
Bidirectional plasticity occurring in V1 during short- and long-term habituation require NMDA receptors in V1. **(A)** Schematic of the experimental set-up in which a Cre recombinase was locally expressed bilaterally in binocular V1 using an AAV viral vector to knockdown the mandatory GluN1 subunit of the NMDA receptor in GluN1-floxed mice. **(B)** Comparison of behavior across blocks for KD group (*n* = 11). Friedman test χ^2^(4) = 4.7, *p* = 0.3. *Post-hoc* analysis of individual comparisons of blocks 1–2: *p* = 0.8, blocks 1–3: *p* = 0.5, blocks 1–4: *p* = 0.3, blocks 1–5: *p* = 0.1. Comparison of behavior across blocks for WT group (*n* = 11). Friedman test χ^2^(4) = 10.8, *p* = 0.03. *Post-hoc* analysis of individual comparisons of blocks 1–2: *p* = 0.1, blocks 1–3: *p* = 0.02, blocks 1–4: *p* = 0.02, blocks 1–5: *p* = 0.02. FDR correction for multiple comparisons. **(C)** Behavioral change over days 1–6 in KD group (*n* = 11), Freidman test χ^2^(5) = 5.9, *p* = 0.3. In WT group (*n* = 11), Freidman test χ^2^(5) = 21.6, *p* = 0.001. FDR correction for multiple comparisons. **(D)** Behavioral response to familiar and novel. Wilcoxon signed-rank test fam vs. nov in KD group: *p* = 0.2, in WT group: *p* = 0.009. FDR correction for multiple comparisons. **(E)** VEP magnitude change from block 1 to block 5 for day 1 to day 6 (*n* = 11 for each group). **(F)** VEP potential magnitude averaged over day 1–6. Comparison over blocks for KD group, Friedman test χ^2^(4) = 0.7, p = 0.9 (*n* = 11). Comparison over blocks for WT group, Friedman test χ^2^(4) = 12.1, *p* = 0.03 (*n* = 11). FDR correction for multiple comparisons. **(G)** VEP magnitude across days 1–6 in knock-down (KD) and wild-type (WT groups). Friedman test for KD group: χ^2^(5) = 15.4, *p* = 0.008 (*n* = 11). Friedman test for WT group: χ^2^(5) = 36.5, *p* < 0.001 (*n* = 11). FDR correction for multiple comparisons. **(H)** Ratio of day 6 VEP magnitude to day 1 VEP magnitude in KD and control group. Wilcoxon signed rank between groups: *p* = 0.04. **(I)** VEP magnitude response to familiar and novel, Wilcoxon signed-rank test fam vs. nov for KD group: *p* = 0.2, for WT group: *p* = 0.003 (*n* = 11). FDR correction for multiple comparisons. Asterisks throughout denote significance (*p < 0.05, **p < 0.01, ***p < 0.001) while ns denotes non-significant.

**FIGURE 4:**
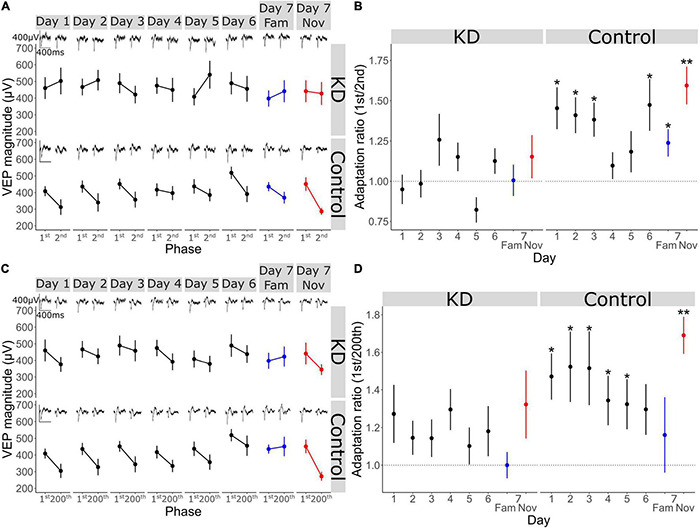
V1 adaptation requires NMDA receptors in V1 across short timescales. **(A)** VEP magnitude in response to the 1st and 2nd phase reversal in GluN1 KD and WT group across all days (*n* = 11) **(B)** adaptation ratio (1st/2nd) across days. Wilcoxon signed-rank test on AR (μ = 1) in KD group on day 1: *p* = 0.6, day 2: *p* = 0.9, day 3: *p* = 0.5, day 4: *p* = 0.4, day 5: *p* = 0.4, day 6: *p* = 0.4, day 7 fam: *p* = 1, day 7 nov: *p* = 0.6. Wilcoxon signed-rank test on AR (μ = 1) in WT group on day 1: *p* = 0.02, day 2: *p* = 0.01, day 3: *p* = 0.02, day 4: *p* = 0.2, day 5: *p* = 0.3, day 6: *p* = 0.03, day 7 fam: *p* = 0.03, day 7 nov: *p* = 0.008. FDR correction for multiple comparisons. **(C)** VEP magnitude in response to the 1st and 200th phase reversal in KD and WT group across all days. **(D)** Adaptation ratio (1st/200th) across days. Wilcoxon signed-rank test on AR (μ = 1) in KD group on day 1: *p* = 0.3, day 2: *p* = 0.3, day 3: *p* = 0.3, day 4: *p* = 0.3, day 5: *p* = 0.5, day 6: *p* = 0.5, day 7 fam: *p* = 0.5, day 7 nov: *p* = 0.4. Wilcoxon signed-rank test on AR (μ = 1) in WT group on day 1: *p* = 0.02, day 2: *p* = 0.02, day 3: *p* = 0.02, day 4: *p* = 0.02, day 5: *p* = 0.05, day 6: *p* = 0.08, day 7 fam: *p* = 0.8, day 7 nov: *p* = 0.008. FDR correction for multiple comparisons. Asterisks throughout denote significance (*p < 0.05, **p < 0.01).

**FIGURE 5:**
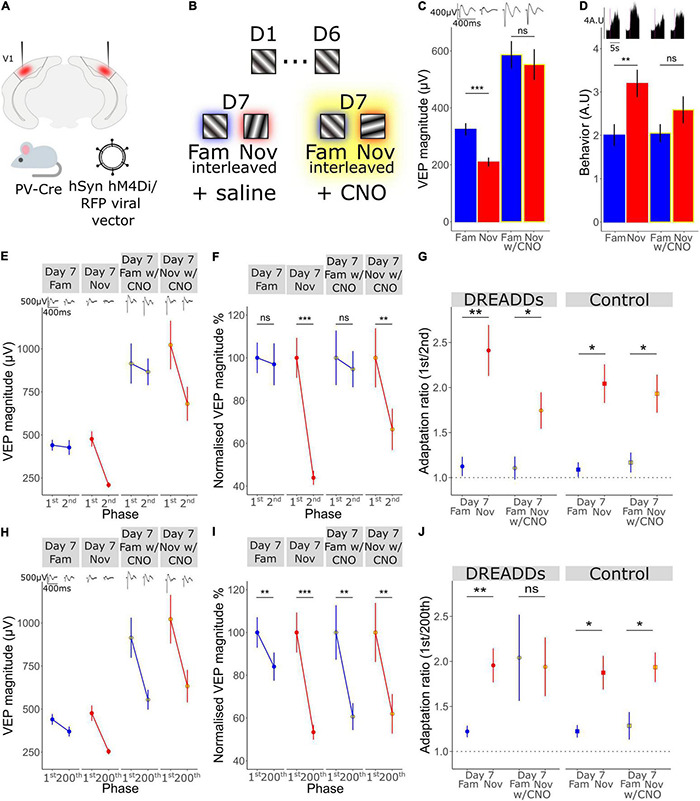
A key role for the activity of Parvalbumin-expressing inhibitory interneurons in long-term familiarity exposes a mechanistic difference between timescales of adaptation. **(A)** Schematic of the experimental set-up in which hM4Di was selectively expressed in parvalbumin-expressing (PV) inhibitory neurons of V1 using an AAV viral vector in PV-Cre mice. **(B)** Schematic of visual presentation protocol in which all mice underwent a standard 6-day SRP protocol before testing response to familiar and novel stimuli during systemic saline injection or CNO application, which were administered prior to presentation of familiar and novel stimuli. **(C)** VEP magnitude in response to familiar and novel stimuli with and without CNO-induced PV+ neuronal inactivation. Wilcoxon signed rank day 7 fam vs. nov: *p* < 0.001. Wilcoxon signed rank day 7 fam vs. nov with CNO: *p* = 0.09. **(D)** Behavioral change in response to familiar and novel stimuli with and without CNO. Wilcoxon signed rank day 7 fam vs. nov: *p* = 0.02. Wilcoxon signed rank day 7 fam vs. nov with CNO: *p* = 0.2. **(E)** VEP magnitude in response to the 1st and the 2nd phase reversal for familiar and novel stimuli with and without CNO. **(F)** VEP magnitude to the 1st and the 2nd phase reversal normalized to the 1st phase reversal in response to familiar and novel stimuli with and without CNO. Wilcoxon signed rank phase 1 vs. 2 on day 7 fam (*n* = 14): *p* = 0.5, day 7 nov: *p* < 0.001, day 7 fam w/CNO: *p* = 0.9, day 7 nov w/CNO: *p* = 0.004. **(G)** Adaptation ratio (1st/2nd) in response to familiar and novel stimuli with and without CNO. Wilcoxon signed rank (*n* = 14): day 7 fam AR vs. day 7 nov AR in DREAADs group: *p* = 0.007; day 7 fam w/CNO AR vs. day 7 nov AR w/CNO in DREAADs group: *p* = 0.02. Wilcoxon signed rank (*n* = 7): day 7 fam AR vs. day 7 nov AR in WT group: *p* = 0.03; day 7 fam w/CNO AR vs. day 7 nov AR w/CNO in WT group: *p* = 0.02. FDR correction for multiple comparisons. **(H)** VEP magnitude in response to the 1st and the 200th phase reversal in response to familiar and novel stimuli with and without CNO. **(I)** VEP magnitude to the 1st and the 200th phase reversal normalized to the 1st phase reversal in response to familiar and novel stimuli with and without CNO. Wilcoxon signed rank phase 1 vs. 200 on day 7 fam: *p* = 0.005, day 7 nov (*n* = 14): *p* < 0.001, day 7 fam w/CNO: *p* = 0.005, day 7 nov w/CNO: *p* = 0.003. **(J)** Adaptation ratio (1st/200th) in response to familiar and novel stimuli with and without CNO. Wilcoxon signed rank day 7 fam AR vs. day 7 nov AR in DREAADs group (*n* = 14): *p* = 0.007; day 7 fam w/CNO AR vs. day 7 nov AR w/CNO in DREAADs group: *p* = 0.8. Wilcoxon signed rank (*n* = 7); day 7 fam AR vs. day 7 nov AR in WT group: *p* = 0.03; day 7 fam w/CNO AR vs. day 7 nov AR w/CNO in WT group: *p* = 0.04. FDR correction for multiple comparisons. Asterisks throughout denote significance (*p < 0.05, **p < 0.01, ***p < 0.001) while ns denotes non-significant.

All procedures were carried out in accordance with the guidelines of the National Institutes of Health and protocols approved by the Committee on Animal Care at the Massachusetts Institute of Technology. Figures 1, 2 are composed of data from male C57B6/J mice (Charles River laboratory international, Wilmington, MA). NMDA knock-down experiments (Figures 3, 4) make use of GRIN*^fl/fl^* mice (B6.129S4-*Grin1^*tm*2S*tl*^*/J—Jackson laboratory). PV+ interneuron inactivation (Figure 5) uses PV-Cre mice (B6;129P2-*Pvalb^TM 1(*cre)Arbr*^*/J—Jackson laboratory). All animals had food and water available *ad libitum* and were maintained on a 12-h light-dark cycle.

### Viral Transfection

In the NMDAR knock-down and PV+ inactivation experiments viral vectors were administered *via* stereotaxic injections into the mice. For the NMDA knock-down, GRIN*^fl/fl^* mice (B6.129S4-*Grin1^*tm*2S*tl*^*/J—Jackson laboratory) underwent surgery at ∼ 1 month. AAV8-hSyn-GFP-Cre (knockdown; UNC viral core) or AAV8-hSyn-GFP (control; UNC viral core; generated by Dr. Bryan Roth’s laboratory) were injected in quantities of 13.5 nl 10 times at depths 600, 450, 300, and 150 μm bellow surface. Each injection was separated by 15 s and after repositioning 5 min was allowed. For the PV+ inactivation experiment, AAV9-hSyn-DIO-HA-hM4D(Gi)-IRES-mCitrine virus (UNC viral core—generated by B. Roth’s laboratory) was injected into PV-Cre or WT-littermates in quantities of 81 nl at depths 600, 450, and 300 μm below surface, including a 5-min delay after repositioning. Viral transfections were performed in both hemispheres and were immediately followed by V1 electrode implantation, outlined below. Following surgery, mice were allowed 3 weeks for full viral expression.

### V1 Electrode Implantation

Mice were anesthetized with an intraperitoneal (i.p) injection of 50 mg/kg ketamine and 10 mg/kg xylazine for surgery. 1% lidocaine hydrochloride anesthetic was injected locally under the scalp and 0.1 mg/kg Buprenex was delivered sub-cutaneously for analgesia. Iodine and 70% ethanol were used to clean the scalp. The skull was cleaned, dried, and scored using a blade. A steel headpost was fixed over the frontal suture using super glue (ethyl cyanoacrylate). Burr holes were drilled 3.1 mm lateral to lambda (to target binocular V1). Tungsten recording electrodes (FHC, Bowdoinham, ME, United States) were implanted 450 μm below surface in both hemispheres. Silver wire reference electrodes were placed in prefrontal cortex bi-laterally.

### Visual Stimuli

Visual stimuli were generated using software developed by Jeff Gavornik.^1^ The display was 20 cm in front of the mouse, and mean luminance was 27 cd/m^2^. Sinusoidal phase reversing gratings were presented full field, reversing at 2 Hz. In most experiments, blocks consisted of 200 phase reversals, each block was presented 5 times interleaved with 30 s of gray screen. Gamma-correction was performed to maintain constant luminance between gratings and gray screen. The 5 blocks were repeated until day 6. On the final day, day 7, the familiar orientation (X°) was pseudo-randomly interleaved (such that no more than 2 blocks of the same orientation were shown in sequence) with a novel orientation (X+90°). Orientations were never within 25° of horizontal. In the PV+ inactivation experiment (Figure 5) 10 blocks were shown. On day 7 familiar (X°) and novel (X°-60°) stimuli were shown. Then CNO was administered at 5 mg/kg *via* intraperitoneal (i.p) injection. After a 15-min wait, the familiar stimulus (X°) was presented with a new novel stimulus (X°+60°).

### *In vivo* Data Acquisition and Analysis

Mice recovered from electrode implantation then underwent 2 days of habituation, followed by the 7-day protocol outlined above. All data was acquired using the Plexon data acquisition system (Plexon Inc., Dallas, TX, United States). Local field potentials (LFP) were collected from V1 in both hemispheres, and piezoelectrical signal was reduced in amplitude and digitized into a third recording channel. Animals were head fixed at the opening of a metal cylinder tube and positioned on a piezoelectric transducer placed under the front paws but touching the metal cylinder. This piezoelectric signal therefore consists mainly of front paw movement but hind paw/whole body movements also contribute to the signal due to vibrations *via* the metal tube. All digital channels were recorded at 1 kHz sampling and run through a 500 Hz low-pass filter. Data was extracted into Matlab using custom software. For the analysis over days, 450 ms traces following stimulus onset were averaged over 1,000 phase reversals (5 blocks × 200 phase reversals). For the across block analysis, traces were averaged over 200 phase reversals. For the within-block analysis (1v2, 1v200), each individual phase reversal was averaged over 5 blocks. VEP magnitude was taken as the minimum microvolt value from 1 to 100 ms following onset subtracted from the maximum microvolt value taken from 75 to 250 ms following onset.

### Statistics

All data is expressed as mean ± SEM and number of animals is represented by n. All statistical analysis is non-parametric due to small n numbers negating true testing of normality. For comparisons between two groups or time points, a paired Wilcoxon signed rank test is used, for adaptation ratio analysis a one-sample Wilcoxon signed rank test is used with a μ of 1. Repeated measures Friedman test is used for analysis across multiple time points within one group. Where multiple tests have been performed, all *p*-values are adjusted using false discovery rate (FDR) correction.

### Data Collection and Use

Data was originally collected by Sam Cooke in Mark Bear’s lab (MIT). Raw data used in Figures 1, 2 was previously published by Kim et al. (2020). Raw data used in Figures 3, 4 was previously published by Cooke et al. (2015), and Figure 5 was published by Kaplan et al. (2016). Extended data analysis was performed on this data which elucidated further phenotypes which are discussed below.

## Results

### Habituation Can Be Observed Within and Across Days in the Same Animal

Visual stimuli were presented over multiple timescales to awake head-fixed mice within a longitudinal experimental design. This approach allowed for investigation into the change in neocortical activity across these different timescales as visual-evoked behavior was concomitantly monitored. Awake mice were head-fixed and viewed full field, oriented, 0.05 cycles/degree, 100% contrast, phase-reversing, sinusoidal grating stimuli while concurrently recording layer 4 local fields potentials (LFPs) with chronically implanted tungsten microelectrodes and behavior using a piezoelectric sensor (Figure 1A). After a 5-min period of gray screen (equivalent luminance to the grating stimuli to follow) to settle the animal into head-fixation, a stimulus of one fixed orientation (X°) was presented at a temporal frequency of 2 Hz for 200 phase reversals, resulting in ∼100 s of continuous stimulus presentation (we describe this as a stimulus block throughout). This block was repeated 5 times with 30-s-long gray screen intervals separating them. Overall, this session lasted approximately 15 min (5 min of gray followed by ∼10 min of stimulus blocks and intervening gray). These sessions, each containing 5 separated blocks, were then repeated once each over 6 days. On the 7th day, 5 blocks of the original orientation (X°) were presented pseudo-randomly interleaved with a novel orientation (X+90°), such that no more than 2 blocks of one orientation were presented in sequence (Figure 1B). This experimental design allowed for analysis of habituation and cortical plasticity across days and within a day.

We found that behavioral habituation occurred both within a day and across days. After the onset of a block of visual stimuli, animals produce a pronounced behavioral response, which we measured using a piezoelectric device and previously termed a vidget (Cooke et al., 2015). Using the vidget, we were able to observe behavioral habituation within a single recording session on day 1 (*n* = 30), when the X° stimulus was novel. The vidget magnitude dropped considerably by the second block and remained low (Figure 1C; Friedman test: *p* = 0.008, Wilcoxon signed-rank on B1–B2: *p* = 0.02, B1–B3: *p* = 0.7, B1–B4: *p* = 0.04, B1–B5: *p* = 0.5; FDR correction for multiple comparisons), indicating the occurrence of short-term habituation on day 1. When averaged over all 5 blocks, the overall magnitude of vidgets was greater on day 1 than on the following days (Figure 1D; Friedman test: *p* = 0.3; Wilcoxon signed-rank on days 1–2: *p* = 0.05, days 1–3: *p* = 0.05, days 1–4: *p* = 0.2, days 1–5: *p* = 0.09, days 1–6: *p* = 0.05; FDR correction for multiple comparisons), indicating the occurrence of long-term habituation. During presentation of blocks of a novel stimulus (X+90°), interleaved with the familiar X° stimulus on the final day, vidgets were increased in magnitude for the novel compared to the familiar stimulus (Figure 1E; Wilcoxon signed-rank test: *p* < 0.001), just as we have described previously (Cooke et al., 2015; Kaplan et al., 2016; Fong et al., 2020).

### V1 Plasticity Accompanying Long- and Short-Term Habituation Occurs in Opposing Directions

Phase-locked LFP responses from layer 4 were averaged together to assess changes in visual-evoked potential (VEP) magnitude within a day and across days (*n* = 33). We found that the changes in VEP magnitude occurred in differing directions dependent upon the timescale. A very clear decrement in VEP magnitude was apparent over the course of 5 blocks of stimulus presentation (∼10 min) within day 1 (Figures 1F,G; Friedman test across blocks on day 1; *p* = 0.01), following the trend of behavioral habituation. This effect became more pronounced after the first day of stimulus presentation (Figure 1F; Friedman test: day 1; *p* = 0.01, day 2–6 *p* < 0.001; FDR multiple comparisons corrected). In contrast, across days there was significant potentiation of VEP magnitude (Figure 1H; Friedman test: *p* < 0.001) and this potentiation was orientation specific, because VEP magnitude was reduced to baseline in response to the novel orientation (Figure 1I; Wilcoxon signed-rank test: *p* < 0.001). Thus, SRP is also present in these animals, just as described previously (Frenkel et al., 2006; Cooke and Bear, 2010). Importantly, a response decrement accompanies short-term habituation, while response potentiation accompanies long-term habituation in the same animals.

### Short-Term Adaptation Occurs Within a Stimulus Block

Next, we wanted to determine whether even shorter timescales of plasticity could be identified within the same experiments, this time focusing on plasticity across a single stimulus block. We averaged VEP magnitude for each of the 200 phase reversals within a block across all 5 blocks on day 1 and across animals (*n* = 33). Over the course of 200 phase reversals (∼100 s) we observed a reduction in the VEP magnitude (Figure 2A). Most notably, there was an immediate reduction from phase 1 to phase 2 (Figures 2A,B; Wilcoxon signed-rank on phase 1–2: *p* < 0.001), followed by a striking rebound over the next few phase reversals. A steadier reduction in VEP magnitude was observed across all 200 phase reversals, culminating in a significant difference between phase reversal 1 and phase reversal 200 (Figures 2A,C; Wilcoxon signed-rank on phase 1–200: *p* = 0.001). Thus, clear evidence is apparent of adaptation within a stimulus block, indicating at least one, and perhaps two additional potential timescales of plasticity to be investigated.

### Short-Term Adaptation Is Modulated by Stimulus Familiarity

Short-term adaptation occurred from both the first to the second and the first to the last phase reversal in a stimulus block when a stimulus was relatively novel on day 1, but did that plasticity persist for highly familiar stimuli? By assessing averaged within-block adaptation over the course of 6 days of long-term observation, we found that adaptation from the first to the second phase reversal was gradually reduced over days (Figure 2D; Wilcoxon signed-rank test on phase 1 vs. 2 on day 1: *p* < 0.001, day 2: *p* < 0.001, day 3: *p* = 0.002, day 4: *p* = 0.008, day 5: *p* = 0.05, day 6: *p* = 0.04; FDR correction for multiple comparisons). Although this adaptation from the first to the second phase reversal lessened as the stimulus became familiar over days, significant adaptation remained and the adaptation ratio (AR) (1st/2nd) was always significantly above 1 (Figure 2E; one sample Wilcoxon signed-rank test on AR (μ = 1) on day 1: *p* < 0.001, day 2: *p* < 0.001, day 3: *p* < 0.001, day 4: *p* < 0.001, day 5: *p* < 0.001, day 6: *p* = 0.002; FDR correction for multiple comparisons). On day 7, there was greater adaptation for the novel stimulus than for the familiar orientation in pseudo-randomly interleaved blocks (Figure 2F; Wilcoxon signed-rank test on phase 1 vs. 2 on day 7 fam: *p* = 0.02, day 7 nov: *p* < 0.001; FDR correction for multiple comparisons) and the AR (1st/2nd) for the familiar stimulus was significantly reduced compared to that in response to the novel stimulus (Figure 2G; Wilcoxon signed rank day 7 fam AR vs. day 7 nov AR: *p* = 0.009) suggesting modulation of adaptation from the 1st to 2nd phase reversal by long-term familiarity.

A more pronounced modulation of adaptation by long-term familiarity was observed for adaptation from the first to the last phase reversal. Adaptation from phase reversal 1 to 200 was no longer significant by day 4 and thereafter (Figure 2H; Wilcoxon signed-rank phase 1 vs. 200 on day 1: *p* = 0.008, day 2: *p* = 0.04, day 3: *p* < 0.001, day 4: *p* = 1, day 5: *p* = 0.4, day 6: *p* = 1; FDR correction for multiple comparisons). In this case, the adaptation ratio (1st/200th) became statistically indistinguishable from 1 by day 4 for the familiar orientation [Figure 2I; one sample Wilcoxon signed-rank test on AR (μ = 1) on day 1: *p* < 0.001, day 2: *p* = 0.001, day 3: *p* < 0.001, day 4: *p* = 0.1, day 5: *p* = 0.006, day 6: *p* = 1; FDR correction for multiple comparisons]. The adaptation from reversal 1 to 200 only returned when a novel orientation was presented on the final day (Figure 2J; Wilcoxon signed-rank phase 1 vs. 200 on day 7 fam: *p* = 1, day 7 nov: *p* < 0.001; FDR correction for multiple comparisons). The AR (1st/200th) for the familiar stimulus was significantly different to that in response to the novel stimulus (Figure 2K; Wilcoxon signed rank day 7 fam AR vs. day 7 nov AR: *p* < 0.001) showing that adaptation from the 1st to 200th phase reversal is strongly modulated by long-term familiarity.

### Both Short-Term and Long-Term Habituation Require NMDA Receptors in V1

Given the critical role of NMDA receptors (NMDAR) in a wide range of plasticity, and a known requirement in SRP and long-term habituation (Frenkel et al., 2006; Cooke et al., 2015), we sought to investigate habituation and accompanying plasticity over shorter timescales after local NMDAR knock-down in V1. Knock-down of NMDAR was achieved by expressing CRE recombinase *via* AAV viral vector injection bilaterally into V1 in a GluN1-floxed (GRIN1 fl/fl) mouse line (Figure 3A), thus knocking down expression of this mandatory subunit for NMDAR only within V1 (*n* = 11 mice). In the control condition, GRIN1 fl/fl littermates were injected with a comparable vector, sharing serotype, promoter and fluorophore, that lacked CRE recombinase (*n* = 11). As we have shown (Figure 1), behavioral habituation occurs both across days and within a day from block 1 to block 5. We found that loss of NMDARs from V1 affects both timescales. Behavioral activity usually drops from the first block to the second and remains low (Figure 1), and we found that to also be true in the WT littermate control mice (Figure 3B; Friedman test for block 1–5: *p* = 0.003, Wilcoxon signed-rank test in WT group B1–B2: *p* = 0.1, B1–B3: *p* = 0.02, B1–B4: *p* = 0.02, B1–B5: *p* = 0.02; FDR correction for multiple comparisons). However, knock-down of NMDARs in V1 prevents the reduction in behavior across blocks (Figure 3B; Friedman test for block 1–5: *p* = 0.3, Wilcoxon signed-rank in KD group B1–B2: *p* = 0.8, B1–B3: *p* = 0.5, B1–B4: *p* = 0.2, B1–B5: *p* = 0.1; FDR correction for multiple comparisons). As we reported previously (Cooke et al., 2015), behavioral habituation from day 1 to day 6 is absent in the KD group (Figure 3C; Friedman test in KD group: *p* = 0.3, in WT group: *p* = 0.001; FDR correction for multiple comparisons). On day 7 there was no difference in the behavioral response between the novel and familiar stimulus in the KD group, whereas in the WT group behavioral activity was higher in response to the novel stimulus (Figure 3D; Wilcoxon signed-rank fam vs. nov in KD: *p* = 0.2, in WT: *p* = 0.009).

### Bidirectional Plasticity Occurring in V1 During Short- and Long-Term Habituation Require NMDA Receptors in V1

Within the same dataset, we now assessed the within-day VEP magnitude reduction that accompanies within-day habituation. The reduction in VEP magnitude across 5 blocks was modest in this dataset and was less apparent in these subjects than in the subjects described in Figure 1 (Figure 3E). Nevertheless, by averaging the block-to-block VEP magnitudes observed during short-term habituation across days, a significant within-day VEP suppression was observed in the GRIN fl/fl littermate control animals (Figure 3F; *n* = 11; Friedman test in control group: *p* = 0.03; FDR correction for multiple comparisons). In contrast, this significant VEP decrement was not observed in the NMDAR KD mice (Figure 3F; *n* = 11; Friedman test in KD group: *p* = 0.9, FDR correction for multiple comparisons), indicating that the within-day reduction in VEP magnitude accompanying short-term habituation requires NMDAR, just as with the habituation itself. As previously reported (Cooke et al., 2015), VEP magnitude potentiation from day 1 to 6, or SRP, is reduced in the knock-down (KD) group compared to control (Figure 3G; *n* = 11; Friedman test in KD group: *p* = 0.008, WT group: *p* < 0.001; FDR correction for multiple comparisons). Comparing the ratio of day 6–1 in the control and KD group shows a significant reduction in this plasticity over days after NMDAR KD (Figure 3H; Wilcoxon signed rank between control and KD day 6/day 1 ratio: *p* = 0.04). On day 7, there was no difference in VEP magnitude between the familiar and novel orientation in the KD group, whereas the VEP magnitude to the novel stimulus in the control group was significantly different (Figure 3I; *n* = 11; Wilcoxon signed-rank fam vs. nov in KD: *p* = 0.2, control: *p* = 0.003; FDR correction for multiple comparisons).

### V1 Adaptation Requires NMDA Receptors in V1 Across Short and Longer Timescales

As we have shown above, short-term adaptation within our paradigm ordinarily occurs from both the 1st to the 2nd phase reversal and the 1st to the 200th phase reversal but disappears as the stimulus becomes familiar (Figure 2). Within the GRIN1 fl/fl dataset, this adaptation was similarly present in the GRIN1 fl/fl controls on day 1 and the subsequent 2 days, eventually becoming non-significant by day 4 and thereafter for highly familiar stimuli [Figures 4A,B; one sample Wilcoxon signed-rank test on AR (1st/2nd) (μ = 1) control group on day 1: *p* = 0.02, day 2: *p* = 0.01, day 3: *p* = 0.02, day 4: *p* = 0.2, day 5: *p* = 0.3, day 6: *p* = 0.03; FDR correction for multiple comparisons]. However, after knock-down of NMDAR in V1, adaptation from the 1st to the 2nd phase reversal was absent on day 1 and all subsequent days [Figures 4A,B; one sample Wilcoxon signed-rank test on AR (1st/2nd) (μ = 1) KD group on day 1: *p* = 0.6, day 2: *p* = 0.9, day 3: *p* = 0.5, day 4: *p* = 0.4, day 5: *p* = 0.4, day 6: *p* = 0.4]. When blocks of stimuli for familiar and novel orientations were presented pseudo-randomly interleaved on day 7, this 1st/2nd reversal adaptation was reduced for familiar but not novel stimuli in the control mice [Figures 4A,B; one sample Wilcoxon signed-rank test on AR (1st/2nd) (μ = 1) on day 7 fam: *p* = 0.03, day 7 nov: *p* = 0.008; FDR correction for multiple comparisons], but not present for either stimulus in the NMDAR KD mice [Figures 4A,B; one sample Wilcoxon signed-rank test on AR (1st/2nd) (μ = 1) KD group on day 7 fam: *p* = 1, day 7 nov: *p* = 0.6]. The same phenotype was present when investigating adaptation from the 1st to the 200th phase reversal. Loss of NMDARs prevented any short-term adaptation expression across all days and stimulus type [Figures 4C,D; one sample Wilcoxon signed-rank test on AR (1st/200th) (μ = 1) KD group on day 1: *p* = 0.3, day 2: *p* = 0.3, day 3: *p* = 0.3, day 4: *p* = 0.3, day 5: *p* = 0.5, day 6: *p* = 0.5, day 7 fam: *p* = 0.5, day 7 nov: *p* = 0.4; FDR correction for multiple comparisons], while it remained present in the control mice over the first 5 days of stimulus presentation, and re-emerged to a novel stimulus on day 7 [Figures 4C,D; one sample Wilcoxon signed-rank test on AR (1st/200th) (μ = 1) control group on day 1: *p* = 0.02, day 2: *p* = 0.02, day 3: *p* = 0.02, day 4: *p* = 0.02, day 5: *p* = 0.04, day 6: *p* = 0.08, day 7 fam: *p* = 0.8, day 7 nov: *p* = 0.008; FDR correction for multiple comparisons]. Thus, short-term adaptation of VEP magnitude in V1 requires the presence of functional NMDAR.

### A Key Role for the Activity of Parvalbumin-Expressing Interneurons in Long-Term Familiarity Exposes a Mechanistic Difference Between Timescales of Adaptation

Previously, we have shown that parvalbumin-expressing (PV+) inhibitory neurons in V1 are critical for the expression of long-term familiarity. We inactivated these neurons using a cell type-specific chemo-genetic approach in which the hM4Di DREADDS receptor was expressed in PV+ neurons of V1, disrupting SRP expression (Kaplan et al., 2016). Therefore, we decided to assess whether these PV+ neurons in V1 are required for the modulation of adaptation by long-term familiarity that we have described in the current study (Figure 2). Bilateral injection of an AAV viral vector into V1 of a PV-Cre mouse to express hM4Di in these cells (Figure 5A) enabled subsequent inactivation of V1 PV+ interneurons after SRP and long-term habituation had been established over 6 days. Specifically, on day 7, familiar (X°) and novel (X+60°) orientations were pseudo-randomly interleaved in a standard design to test for selective SRP/habituation to the familiar orientation. After this, mice were systemically injected (i.p.) with clozapine-n-oxide (CNO), which binds to hM4Di to inactivate expressing neurons, before re-testing response to blocks of the familiar and a new novel stimulus (X-60°) to assess modulation of adaptation by long-term familiarity (Figure 5B). Prior to inactivation of PV+ neurons, VEP magnitude was significantly potentiated in response to the familiar stimulus and therefore significantly greater in magnitude than response to the novel stimulus (Figure 5C; Wilcoxon signed-rank day 7 fam vs. nov: *p* < 0.001; FDR correction for multiple comparisons). However, as we have reported previously (Kaplan et al., 2016), after inactivation of PV+ interneurons, there was no significant difference in VEP magnitude in response to familiar and novel stimuli (Figure 5C; Wilcoxon signed-rank day 7 fam vs. nov w/CNO: *p* = 0.09; FDR correction for multiple comparisons). It is important to note that after inactivation of PV+ interneurons, the general VEP magnitude was higher due to the loss of inhibition in the cortex. The inactivation of V1 PV+ inhibitory neurons also impaired behaviorally manifest novelty detection as the behavioral response to a novel stimulus was significantly greater than the response to the familiar stimulus before inactivation of PV+ neurons (Figure 5D; Wilcoxon signed-rank day 7 fam vs. nov: *p* = 0.02; FDR correction for multiple comparisons), but was suppressed after inactivation of these neurons and no longer different during PV+ inactivation (Figure 5D; Wilcoxon signed-rank day 7 fam vs. nov w/CNO: *p* = 0.2; FDR correction for multiple comparisons).

As we have shown in the current study, short-term adaptation from the first to the second phase reversal progressively reduces as the stimulus becomes familiar and is selectively suppressed on day 7 to highly familiar stimuli, but not novel stimuli (Figure 2). Here we show that, although VEP magnitude generally increases, inactivation of PV+ interneurons had no effect on the modulation of 1st/2nd phase reversal short-term adaptation (Figures 5E–G). Strong adaptation from the first to the second phase reversal was absent when the stimulus was familiar and present when the stimulus was novel, regardless of whether PV+ neurons were inactivated. This observation is most clear when we normalize to the magnitude of the first phase reversal in order to remove the confound of increased overall response after PV+ inactivation [Figure 5F; normalized to the first phase reversal; Wilcoxon signed rank phase 1 vs. 2 on day 7 fam: *p* = 0.5, day 7 nov: *p* < 0.001, day 7 fam w/CNO: *p* = 0.9, day 7 nov w/CNO: *p* = 0.004 (*n* = 14)]. The adaptation ratio (1st/2nd) was significantly different between the familiar and the novel stimulus both before and after PV+ neuronal inactivation [Figure 5G; Wilcoxon signed rank on day 7 fam AR vs. day 7 nov AR: *p* = 0.007, Wilcoxon signed rank on day 7 fam w/CNO AR vs. day 7 nov w/CNO AR: *p* = 0.02 (*n* = 14)]. Thus, inactivation of PV+ interneurons does not affect the short-term adaptation from the 1st to the 2nd phase reversal, nor its suppression by long-term familiarity.

Strikingly, the adaptation from the first to the last phase reversal of a stimulus block follows a different pattern. While adaptation is suppressed by familiarity on day 7 but present for the novel stimulus before PV+ neuronal inactivation (Figures 5H–J), it is strongly apparent for both familiar and novel stimuli during PV+ neuronal inactivation [Figure 5I; normalized to the first phase reversal; Wilcoxon signed rank phase 1 vs. 200 on day 7 fam: *p* = 0.005, day 7 nov: *p* < 0.001, day 7 fam w/CNO: *p* = 0.005, day 7 nov w/CNO: *p* = 0.003 (*n* = 14)]. The adaptation ratio (1st/200th) is significantly different for familiar and novel stimuli before PV+ inactivation [Figure 5J; Wilcoxon signed rank on day 7 fam AR vs. day 7 nov AR: *p* = 0.007 (*n* = 14)]. After application of CNO the AR is equivalent for both the familiar and novel stimuli (Figure 5J; Wilcoxon signed rank on day 7 fam w/CNO AR vs. day 7 nov w/CNO AR: *p* = 0.8). Therefore, the modulation of the short-term adaptation from the 1st/200th phase reversal by familiarity is not present after inactivation of PV+ interneurons, which differs from the effect on adaptation from the 1st/2nd phase reversal, indicating two mechanistically distinct processes.

## Discussion

**FIGURE 6:**
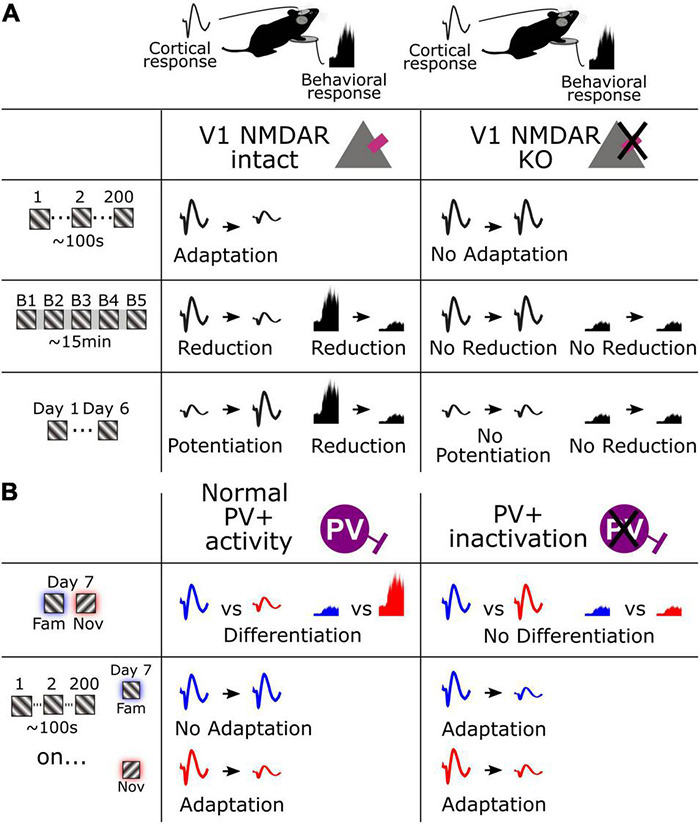
Schematic summarizing fundamental cortical and behavioral changes across multiple timescales. **(A)** Cortical and behavioral changes over seconds, minutes, and days (left), and the result of V1 NMDAR KO on these changes. **(B)** Cortical and behavioral changes in response to a familiar and novel stimulus and the associated adaptation (left), and the result of PV+ interneuron inactivation on these changes.

In the current study we have identified multiple timescales of visual response adaptation that occur during habituation in mice. We have expanded on our previous characterization of stimulus-selective response potentiation (SRP), a form of long-term cortical response potentiation that occurs concomitantly with long-term habituation, to reveal that the reverse effect of response decrement coincides with short-term habituation. Moreover, we have identified shorter-term forms of adaptation that occur over seconds. We also reveal that the NMDA receptor serves as a key molecular mechanism shared by all these forms of plasticity (Figure 6A). In addition, we show that these various forms of plasticity are not isolated phenomena, because short-term adaptation and SRP over days clearly interact, such that adaptation no longer occurs for highly familiar stimuli. We also demonstrate that this suppression of adaptation across hundreds of stimuli by long-term familiarity is gated by the activity of PV+ inhibitory interneurons in V1 because inactivating these neurons causes short-term adaptation to re-emerge to highly familiar stimuli (Figure 6B). Finally, we make the important observation that the fastest form of adaptation that we have measured, occurring within a second of stimulus presentation, remains suppressed for familiar stimuli even after inactivation of PV+ interneurons, indicating that there may be at least two mechanistically separable timescales of adaptation present within our paradigm. Thus, we have revealed a multitude of forms of cortical plasticity that can be assessed in passively viewing mice to gain a deeper understanding of the processes of habituation.

The longest-term form of plasticity we have described here is already well characterized: potentiation of the VEP in layer 4 over days is described as SRP due to its high degree of stimulus-selectivity (Frenkel et al., 2006; Cooke and Bear, 2010) and it occurs concurrently with long-term behavioral habituation (Cooke et al., 2015; Kaplan et al., 2016; Fong et al., 2020; Finnie et al., 2021), just as we further confirm here. Despite the clear reliance of SRP and accompanying habituation on V1 NMDA receptors, selective knock-down of NMDARs in excitatory neurons of layer 4, the locus where SRP is manifest, does not impair SRP or accompanying habituation (Fong et al., 2020). This observation indicates that the potentiation is an echo of plasticity occurring elsewhere in V1, or in a different cell type within layer 4. Therefore, the direct strengthening of synapses at thalamocortical inputs to layer 4 now seems an unlikely explanation for SRP. Although local field potentials are thought to primarily report synaptic activity rather than action potentials (Katzner et al., 2009; Buzsáki et al., 2012), potentiation of VEP magnitude may reflect a loss of shunting inhibition that allows an increased synaptic response to thalamic input, rather than a potentiation of the synaptic input itself. We have previously shown that parvalbumin-expressing (PV+) inhibitory interneurons, which provide this powerful shunting inhibition, show reduced activity over days as the stimulus becomes familiar during SRP (Hayden et al., 2021). In addition, cell-specific interventional approaches reveal that a normal range of activity in PV+ neurons is required for differential response to familiar or novel stimuli after SRP, either cortically or behaviorally (Kaplan et al., 2016). Thus, it seems likely that SRP reflects a loss of PV+ inhibition. How this contributes to a decrement in behavior, as is observed in the concomitant long-term habituation, remains unclear (Montgomery et al., 2021). One possible arrangement is that increased cortical output recruits another form of inhibition to suppress behavioral output. This arrangement would accord with the comparator model of habituation, in which long-lasting memory is formed in the cortex through elevated synaptic activity that enables recognition of familiarity and suppresses output through feedforward inhibition, as suggested by Sokolov (1963) and others (Konorski, 1967; Wagner, 1981). To confirm that SRP conforms to this model will require measurement of V1 output from the deeper layers of neocortex, with the prediction that this activity is suppressed by superficial layers as they exhibit potentiation. It will also be critical to identify the inhibitory intermediary that leads to this cortical output. One strong candidate for this inhibitory suppression has recently emerged (Pluta et al., 2019).

The behavioral response decrement over the course of minutes, reflecting habituation over an intermediate time-scale, has been investigated by others (Sanderson and Bannerman, 2011). The reduction in VEP magnitude that coincides with this within session habituation has not formally been described by us previously. Our observations of a decrement in VEP magnitude are notable because of the striking contrast with SRP, which coincides with a similar reduction in behavior in the same animals, but in that case over days (Figure 1). Visual cortical activity decreases during repetitive presentation of natural movies (Deitch et al., 2021), suggesting that this reduced activity can occur in response to multiple different types of visual stimuli, and the well-documented phenomenon of mis-match negativity, in which novel oddball stimuli evoke increased magnitudes of event-related potentials (ERP) relative to repetitions of increasingly familiar stimuli, occurs across similar timescales (Näätänen et al., 2007; Garrido et al., 2009). In a similar paradigm to ours, thalamic activity has been observed to increase over ∼30 min (Durkin and Aton, 2019), and it remains possible that the plasticity they have observed is, through some unidentified inversion, the origin of cortical decrement and behavioral habituation. However, the reliance of both VEP decrement and concomitant habituation on NMDARs within V1 strongly suggests that this is not the case (Figure 3). Dual recordings of thalamic and cortical neurons may be required to resolve the origins of these effects, and targeted interventions in the thalamus may also prove informative. Investigation of changes over the course of minutes in response to both a familiar and novel grating (currently not possible due to the interleaving of these stimuli) would elucidate if this reduction of cortical activity is indiscriminate to the type of visual stimulus being shown or is also orientation specific, indicating cortical plasticity that is potentially very similar to the familiarity effect observed leading up to mismatch negativity. Recent work has shown that mismatch negativity depends upon activity of the somatostatin-expressing (SST+) inhibitory interneurons (Hamm and Yuste, 2016), suggesting that modification of SST+ inhibition may account for our observations. This class of interneurons primarily target dendrites of excitatory cells and PV+ interneurons (Cottam et al., 2013; Pfeffer et al., 2013; Xu et al., 2013; Rikhye et al., 2021) and they have been shown to be strongly influenced by stimulus familiarity (Kato et al., 2015; Makino and Komiyama, 2015; Hayden et al., 2021). Inhibition on the dendrites of excitatory neurons, where the majority of synaptic contacts are made, may contribute to reduced synaptic activity during habituation (Natan et al., 2015), or these cells may influence the activity of PV+ neurons to mediate the reduction in V1 response, as they are known to do in layer 4 (Xu et al., 2013). It would be informative to measure the activity of these inhibitory neurons in layer 4 of V1 across this timescale and more informative still to monitor inhibitory responses in principle excitatory neurons during this within-session habituation. Given the dependency of the phenomenon that we have described on NMDARs, one intriguing hypothesis is that excitatory synapses onto SST+ neurons are potentiated during repeated stimulus presentation. Knocking down the NMDAR expression within these cells would test this hypothesis. It also remains possible that other types of inhibition are increasingly engaged to produce habituation, as has recently been hypothesized (Ramaswami, 2014). In line with the NMDAR dependence of the reduced behavioral responses, again, this process may involve synaptic depression of excitatory synapses within V1. Much further work is required to investigate the underlying mechanisms of this intermediate form of behavioral and cortical response adaptation.

Over even shorter timescales of seconds, the VEP adaptation that we observe here within continuous blocks of stimulation is a commonly reported phenomenon (Chung et al., 2002; Beierlein et al., 2003; von der Behrens et al., 2009; Cruikshank et al., 2010). The most parsimonious explanation for response decrement is that it reflects a depression of excitatory synapses within the canonical excitatory pathway of V1 through a process of adaptive filtration, which is perhaps the dominant theory of habituation (Horn, 1967; Groves and Thompson, 1970). This depression could potentially occur through Hebbian depression mechanisms (Lee et al., 1998) at excitatory synapses within the cortex (Chen et al., 2015), or the thalamus (Li et al., 2003), or through short-term effects on synaptic release (Moulder and Mennerick, 2006). That the origin of response depression is cortical is supported by its reliance on V1 NMDARs. Specifically, we show that both the adaptation from the 1st to the 2nd phase reversal (0.5 s), and the adaptation from the 1st to 200th phase reversal (100 s) is impaired by a loss of NMDAR expression in V1 (Figure 3). This somewhat surprising finding implicates the occurrence of a Hebbian form of plasticity that is at least induced post-synaptically at short timescales (Bliss and Collingridge, 1993). Additionally, we have made the intriguing additional observation that a loss of activity in PV+ neurons after chemo-genetic inactivation re-instates short-term adaptation even to highly familiar stimuli (Figure 4). The immediate conclusion from this observation is that short-term adaptation does not rely in any way on inhibition mediated by PV+ neuronal activity, in striking contrast to long-term familiarity. The reinstated short-term adaptation may therefore arise from the cortex responding to a familiar stimulus as if it were novel. Alternatively, it remains possible that the loss of adaptation with long-term familiarity arises from a gradual reduction in PV+ mediated inhibition through the course of a stimulus block that perfectly matches excitatory synaptic depression. Inactivation of PV+ neurons would remove this gradual effect and expose the depression occurring at those excitatory inputs. Using calcium imaging, we have previously observed the gradual loss of PV+ neuronal engagement across phase reversals for familiar but not novel stimuli, so this remains a plausible arrangement (Hayden et al., 2021). Interestingly, using a similar method in excitatory neurons we have also previously reported a perplexing mismatch with the electrophysiological measurements of SRP: when measuring VEP magnitude or peak unit firing rate, a pronounced potentiation is observed (Cooke et al., 2015), while a reduction of signal is observed with calcium imaging (Kim et al., 2020). In the current study we have added to that conundrum, as we reveal short-term adaptation across seconds that is limited to novel stimuli (Figure 2), while we previously revealed a similar effect with calcium imaging but limited to familiar stimuli (Kim et al., 2020). The only likely explanation for these curiously mismatched observations is that our electrophysiological methods have detected a fast phasic effect which is potentiated by familiarity over days and diminished to novel stimuli over seconds, while the calcium sensors detect a more sustained diminishment of calcium flux as a result of familiarity over either time-course. Further experiments comparing phasic and drifting gratings or using intracellular electrophysiology may be informative in this regard. It will also be interesting to use calcium imaging to assess the intermediate timescale that we have reported here which occurs from block to block over minutes within a session (Figure 1), to determine if the mismatch between the two methods persists even across this timescale. Our prior study indicates that for this timescale, at least, findings with electrophysiology and calcium imaging will align (Kim et al., 2020).

The storage and retrieval of familiarity plays a major role in reserving energy and attention for only those stimuli that are most pertinent to a task or context and is therefore critical for survival and wellbeing. Understanding how these apparently simple forms of learning and memory are implemented is a greater challenge than expected and there appear to be multiple solutions to the same problem, some of which engage feedforward plasticity, others which engage inhibitory systems and more complicated circuitry. These various mechanisms may all play out within one structure but across different timescales. In this study, we have revealed the measurement of multiple mechanistically distinct forms of plasticity occurring in the same animals across seconds, minutes, and days of repeated stimulus presentation, providing great potential to gain a deep understanding of a foundational set of learning and memory processes. We have monitored these changes using LFP recordings, suggesting that much of the observed phenomenology is likely to translate to non-invasive electroencephalogram (EEG) recordings, providing future potential for translation into human subjects, where forms of plasticity such as mismatch negativity have already been described (Näätänen et al., 2007).

## Data Availability Statement

The original contributions presented in the study are included in the article/supplementary material. Further inquiries can be directed to the corresponding author/s.

## Ethics Statement

The animal study was reviewed and approved by the Committee on Animal Care at the Massachusetts Institute of Technology.

## Author Contributions

SC acquired all the data and participated in experimental design. FC analyzed the data. FC and SC interpreted the data and wrote the manuscript. Both authors contributed to the article and approved the submitted version.

## Conflict of Interest

The authors declare that the research was conducted in the absence of any commercial or financial relationships that could be construed as a potential conflict of interest.

## Publisher’s Note

All claims expressed in this article are solely those of the authors and do not necessarily represent those of their affiliated organizations, or those of the publisher, the editors and the reviewers. Any product that may be evaluated in this article, or claim that may be made by its manufacturer, is not guaranteed or endorsed by the publisher.

## Footnotes

1
https://github.com/jeffgavornik/VEPStimulusSuite
